# Short Overview of ROS as Cell Function Regulators and Their Implications in Therapy Concepts

**DOI:** 10.3390/cells8080793

**Published:** 2019-07-30

**Authors:** Lidija Milkovic, Ana Cipak Gasparovic, Marina Cindric, Pierre-Alexis Mouthuy, Neven Zarkovic

**Affiliations:** 1 Laboratory for Oxidative Stress, Division of Molecular Medicine, Rudjer Boskovic Institute, Bijenicka 54, 10000 Zagreb, Croatia; 2 Laboratory for Molecular Pathology, Department of Pathology and Cytology, University Hospital Centre Zagreb, Salata 10, 10000 Zagreb, Croatia; 3 National Institute for Health Research Oxford Musculoskeletal Biomedical Research Unit, Botnar Research Centre, Nuffield Department of Orthopaedics, Rheumatology and Musculoskeletal Sciences, University of Oxford, Oxford OX3 7LD, UK

**Keywords:** reactive oxygen species (ROS), redox signaling, cellular processes, physiology, cancer, metabolism, therapy

## Abstract

The importance of reactive oxygen species (ROS) has been gradually acknowledged over the last four decades. Initially perceived as unwanted products of detrimental oxidative stress, they have been upgraded since, and now ROS are also known to be essential for the regulation of physiological cellular functions through redox signaling. In the majority of cases, metabolic demands, along with other stimuli, are vital for ROS formation and their actions. In this review, we focus on the role of ROS in regulating cell functioning and communication among themselves. The relevance of ROS in therapy concepts is also addressed here.

## 1. Introduction

The understanding of the role of reactive oxygen species (ROS) in our cellular functioning has changed over the last few decades, from the first perception of ROS as harmful and unwanted byproducts of oxidative stress [1] (something else formerly considered to be unwanted) to realizing their significance in redox signaling. The notion of oxidative stress as a harmful process leading to the formation of ROS has since been upgraded, and different subclasses of oxidative stress are generally classified into eustress and distress [2,3]. The formation of lower levels of ROS that act as redox signaling messengers needed for the normal physiological functioning of cells is denoted as good stress, or eustress. In contrast, the overwhelming accumulation of ROS manifested as a loss of signaling ability and unspecific damage of cellular macromolecules which contributes to different pathologies is denoted as bad stress, or distress. Hence, the consideration of antioxidants as “defenders” that are able to amend these harmful ROS-induced events emerged. Unfortunately, clinical data has not supported this notion due to controversy regarding the obtained results, suggesting that redox-sensitive pathophysiology needs further research. The current knowledge addressing these issues was recently comprehensively reviewed by the Cost Action BM1203 (EU ROS) members [4]. Therefore, a better understanding of both terms, especially eustress, regarding ROS-specific targets and the conditions in which they occur is a constant pursuit in the field of redox biology. The answers to the raised questions would also improve our knowledge of clinically reliable biomarkers of oxidative stress [5].

In this short review, we endeavor to highlight the importance of ROS in cellular functioning within each cell and among them, which is intertwined with metabolism and therefore cell/tissue specific. We further explore the notion that ROS are messengers in the transduction of certain cues (e.g., metabolic and environmental) involving ROS-activated transcription factors and other proteins, thus affecting diverse signaling pathways which, ultimately, determine cell fate. Our aim is to point out that cells, depending on the cues, reorganize their processes, mainly metabolism, to produce ROS that, because they are signaling molecules, will thereby affect cell fate. In this way, we highlight ROS as instruments and tools that enable cells and tissues to function thus as regulators of cell fate rather than as signaling molecules. Additionally, the ROS-related actions of some drugs and therapy strategies are mentioned.

## 2. ROS Sources and Functions

**Figure 1:**
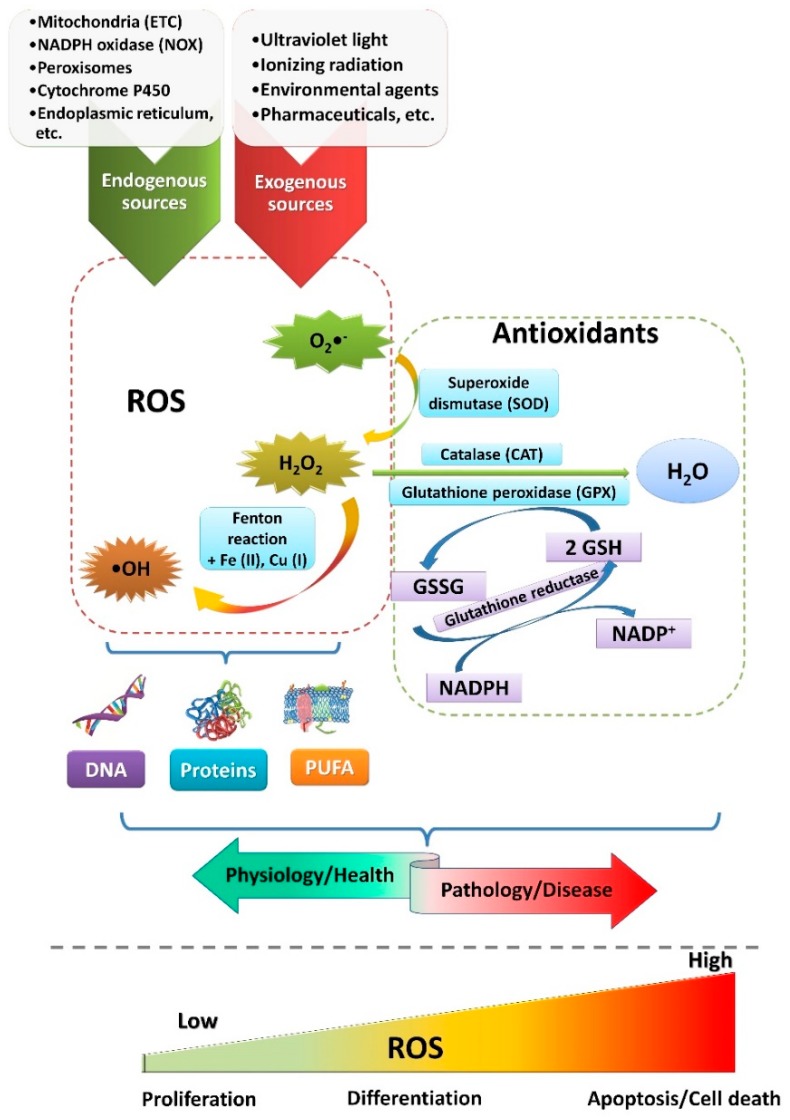
Generation of reactive oxygen species (ROS) and their impact on cells. This is a simplified scheme representing exogenous (e.g., UV light, ionizing radiation, pharmaceuticals, etc.) and endogenous sources (e.g., electron transport chain in mitochondria, NADPH (nicotinamide adenine dinucleotide phosphate) oxidases, peroxisomes, endoplasmic reticulum, etc.) of ROS formation. Cells have evolved antioxidative mechanisms (e.g., glutathione (GSH), superoxide dismutase (SOD), catalase (CAT), glutathione peroxidase (GPX), etc.) that fine-tune ROS levels, ensuring normal cellular functioning. Thus, depending on the levels, ROS affect different transcription factors, enzymes, and/or other proteins, inducing signaling pathways that assure proper functioning and, in general, health. In contrast, higher levels of ROS lead to irreversible damage of macromolecules, thus eventually leading to disease. Again, depending on the levels, ROS impact cellular functions such as proliferation, differentiation, and apoptosis.

Oxygen, which is essential for life, is also, to some minor extent, converted to ROS. The most common representatives of ROS are superoxide anions, hydrogen peroxide, and hydroxyl radicals, in addition to many others. Diverse intracellular sources such as mitochondria, NADPH (nicotinamide adenine dinucleotide phosphate) oxidases (NOX), cytochrome P450, endoplasmic reticulum, peroxisomes, lysosomes, and others produce ROS [6]. Exogenously, ROS are also formed under the influence of ultraviolet light, ionizing radiation, and xenobiotics [7] (Figure 1). As mentioned, ROS are both essential for normal cellular functioning but also detrimental if produced in excess (the amount considered excessive is cell/tissue-type specific and depends on the specific ROS that accumulate; e.g., the hydroxyl radical is the least tolerable due to its high damaging ability).

To cope with elevated levels of ROS, cells have evolved antioxidative machinery comprising enzymes and nonenzymatic antioxidants (e.g., glutathione (GSH), uric acid, melatonin, vitamins C and E, polyphenols, etc.) [7,8]. Enzymes involved in the first line of defense against ROS include superoxide dismutase (SOD), catalase (CAT), and glutathione peroxidase (GPX) (Figure 1). SOD catalyzes the conversion of superoxide anions to hydrogen peroxide, which can be further reduced to water in a reaction catalyzed by CAT or by GPX. The reaction involving GPX requires oxidation of GSH and the formation of glutathione disulfide (GSSG), which is reduced back again to GSH by glutathione reductase [9,10]. Yet, the presence of trace metals can catalyze the formation of more destructive hydroxyl radicals from hydrogen peroxide via the Fenton reaction (Figure 1). Therefore, the production of ROS is in delicate balance with the described antioxidative defense mechanisms. If this balance is disturbed, the accumulation of ROS could become detrimental, leading to diverse pathologies such as neurodegenerative diseases [11], atherosclerosis [12], cancer [13], and others.

Redox signaling, as a term, mainly includes reversible modification, either oxidation or covalent adduct formation, of specific target proteins, allowing further translation of a signal which will ultimately determine cellular fate. Cysteine residues are the most susceptible to oxidation. Yet, notably, not all cysteine residues of a target protein are susceptible to modification but rather specific ones. Location (vicinity to ROS) and kinetics determine their specificity [14]. While signaling abilities of superoxide anions and hydroxyl radicals are considered modest or even nonexistent due to the lower stability and unspecific reactivity, respectively, hydrogen peroxide is depicted as the most important signaling molecule involved in redox signaling (additional reading suggestions [13,15,16]).

Better stability and the ability to pass through cellular membranes, thus diffusing away from its place of origin, ensure the superiority of hydrogen peroxide compared with, for example, superoxide anion. The fine-tuning of hydrogen peroxide levels within and between cells and extracellular space requires efficient transport across the membrane. Among a family of transmembrane water channels, named aquaporins, specific isoforms (“peroxiporins”) are responsible for hydrogen peroxide transport [17,18,19]. Yet, redox biology is rather complex and, also, other molecules contribute to redox signaling, such as hydroperoxides [14], 4-hydroxynonenal (HNE; ROS-induced lipid peroxidation product) [20], as well as peroxiredoxins and thioredoxins, exhibiting not merely an antioxidant role [21]. In addition, many target proteins (such as several kinases, transcription factors, phosphatases, etc.) are involved in redox signaling [22]. Therefore, the causally consequential interplay between ROS (as well as other messenger molecules) and metabolism ensures tight regulation of redox signaling, thus controlling cellular functions, from proliferation to differentiation and apoptosis.

Diverse signaling pathways are activated by ROS, thus determining cellular fate (see [9,23]). ROS activates the NRF2/KEAP1 (nuclear factor erythroid 2 (NF-E2)-related factor 2/Kelch-like ECH-associated protein 1) pathway, which serves as a master regulator of ROS levels. In particular, ROS-induced modification of specific cysteine residues of KEAP1 occurs, leading to disruption of the KEAP1-dependent degradation of NRF2, translocation of NRF2 to the nucleus, and its activation of specific cytoprotective genes [15,24]. Additional pathways regulated by ROS include nuclear factor-κB (NF-κB; of significance in inflammation and immunity) [25,26], phosphoinositide 3-kinase (PI3K)/AKT [27], MAPK (mitogen-activated protein kinase), as well as others [23]. Therefore, depending on the stimuli, ROS activate diverse targets, initiating pathways involved in growth promotion and survival (including autophagy) [28] or apoptosis [29].

## 3. Cell/Tissue-Specific Roles of ROS in (Patho)physiology

The importance of ROS in the functioning of diverse cell types and, consequently, in tissues is immense. Here, we provide just a few examples that highlight ROS as important regulators/cellular tools for reaching specific cell/tissue functions. The recent notion of ROS-directed evolutionary advances of multicellular organisms substantiates this claim. Its main feature is the formation of cytoskeleton-based membrane protrusions that enable intercellular communication and transport of macromolecules [30]. Therefore, ROS not only guide tissue maintenance, but they are also important in the earliest phases of embryonic development starting from fertilization [31,32], as it seems that calcium signaling induces the formation of ROS either by activation of Udx1, a dual NOX [33], or in mitochondria [34], which then blocks polyspermy and directs zygote cleavage.

### 3.1. Stem Cells

Stem cells are important for embryonal and fetal development and in the maintenance of adult tissue homeostasis. Their main features are self-renewal and differentiation into various types of cells, the potential of which depends upon the type of stem cell. Whether stem cells will remain quiescent or progress to self-renewal or differentiation is strongly affected by metabolic changes. These changes are, in a feedback loop, governed by ROS that through activation of various but stimuli-specific target proteins, such as transcription factors, kinases, and phosphatases, further orchestrate cellular processes, thus regulating cell cycle progression (proliferation or arrest), differentiation, quiescence, senescence, or apoptosis [35]. Therefore, ROS were proposed to function as a stem cell rheostat, translating different cues (metabolic and environmental) to coordinate various cellular responses [36]. To which cellular process ROS will direct a stem cell is highly stem-cell-type specific and intertwined with metabolism. For example, self-renewal in embryonic stem cells is a rapid process due to the shortened G1 cell cycle phase. For that reason, it needs much energy and many building blocks, which are preferentially achieved by glycolysis and the pentose phosphate pathway (PPP), respectively [37]. Of note is the fact that different stem cells vary in ROS levels. While a low level of ROS selects more potent hematopoietic stem cells [38], accumulation of ROS controls proliferation and self-renewal in neuronal stem cells [39]. In mesenchymal stem cells, ROS seem to have a double role. Although the undifferentiated state is correlated with lower levels of ROS and the buildup of antioxidative enzymes, mesenchymal stem cells need to upregulate ROS for proliferation and self-renewal [40]. A recent review paper focusing on the cell fate decisions of different types of stem cells emphasized the importance of metabolism, ROS, pH, as well as cell morphology and highlights—indeed, that “the link between the metabolic cue and the cell fate decision is reactive oxygen species” [41].

### 3.2. Immune Cells

The primary role of immune cells is the defense of the host organism against infection induced by invading pathogens. Different types of cells mediate this defense with distinct responses. Phagocytic cells (mainly neutrophils and macrophages) are engaged in an innate immunity response which is nonspecific, while lymphocytes mediate an adaptive immune response. ROS are important for both innate and adaptive immunity [9]. The first beneficial role described attributed to the generation of ROS was phagocytic elimination of microorganisms. Phagocytic recognition of a specific pattern of a microorganism initiates NOX2 production of ROS that will ultimately kill this microorganism. Convincingly, patients suffering from chronic granulomatous disease (CGD) have a heightened susceptibility to infection due to the mutations in NOX2 [42]. ROS were also suggested not only to coordinate the movement of the polymorphonuclear leukocytes towards pathogens and their retention at the site during initiation of inflammation but also to terminate inflammation [43]. In adaptive immunity, ROS mediate activation of T cells and have been suggested to have an immunosuppressive role. T-cell activation also mandates the help of accessory cells to some extent. Induction of regulatory T (Treg) cells by macrophage-derived ROS was shown to suppress other T cells also through ROS. Moreover, localized production of ROS directs the commitment of the Treg lineage, while scavenging ROS decrease the Treg/T-cell effector balance [43,44].

### 3.3. Central Nervous System (CNS)

High energy demands, high oxygen consumption, high levels of iron and polyunsaturated fatty acids, low antioxidative protection, and a delicate blood supply make the CNS, in particular the brain, an easy target of oxidative damage [45]. ROS have been implicated in many pathological conditions, such as neurodegenerative disorders, stroke, and brain tumors [46], due to the high levels found, but their physiological roles have been mainly neglected until recent years.

Inevitably, there is a link between ROS formation and the metabolism of cells. Metabolic processes in neurons, glial cells, and endothelial cells, although largely different and variable, are complementary to assure proper functioning of the brain [47]. While oxidative phosphorylation (OXPHOS) is the primary energy-obtaining pathway in neurons, astrocytes rely mostly on glycolysis. Such conversion of glucose to lactate also serves as an energy source for neurons in a process known as metabolic coupling [48]. This fine astrocyte–neuron cooperation includes coordination of glucose metabolism and antioxidative protection, especially for neurons with diminished levels of antioxidants [49,50]. ROS produced by metabolic processes modulate the physiological processes of neuronal development, regulation of neuronal polarization, connectivity, and plasticity [51,52]. NOX have been proposed as the main source of ROS required for regulation of processes in the brain. For example, stemness and proliferation of neural progenitor cells were shown to be under strict control of NOX2-ROS production and PI3K/AKT signaling [39]. The premise that ROS are maintenance regulators of neuronal progenitor cells was also supported by a NOX2 knock-out mouse model, which revealed a decreased number of proliferating neural stem cells and reduced adult neurogenesis [53].

Recently, mitochondrial ROS emerged as predominant for controlling microenvironmental cues, highlighting astrocytes as the most sensitive sensors, regulators, and protectants of neural functions. Higher mitochondrial ROS levels in astrocytes, associated with the more loose organization of the mitochondrial transport chain [54], are responsible for more robust antioxidative protection. Vicente-Gutierrez et al. have linked mitochondrial and extracellular ROS production, showing that these higher physiological mitochondrial ROS levels in astrocytes keep NRF2 constitutively active, which consequently attenuates NOX1 and NOX2 expression, thus repressing the release of extracellular ROS. Moreover, mitochondrial ROS-induced NRF2 activity maintains sufficient GSH levels in the extracellular matrix, allowing astrocytes to oversee the redox balance in neurons [55]. Aside from the beneficial roles of ROS in the regulation of physiological processes, the excess of ROS that could not be hampered by antioxidative mechanisms contributes to macromolecular damage and the onset of various CNS diseases.

### 3.4. Other Examples of Cell/Tissue-Specific Roles of ROS

In the kidney, ROS are important for regulation of both physiological and pathological processes. While they are needed for protection and restoration of normal kidney functioning, during kidney damage, such as ischemia-reperfusion injury, ROS recruit neutrophils and also trigger NRF2 signaling to bust antioxidative protection (reviewed in [56]). In addition, endothelial NOX2 and NOX4 were shown to be sources of hydrogen peroxide, which acts as a regulator of the vessel wall tension, thus ensuring proper functioning of kidney vasculature [57]. Moreover, NOX4-mediated production of hydrogen peroxide might have an antiatherosclerotic function as well [58].

Another example of an ROS-mediated cell-specific role is platelet generation. It includes different stages, starting from the commitment of hematopoietic stem cells into the megakaryocytic lineage, megakaryocytic progenitor proliferation, differentiation and maturation, cell apoptosis, and platelet release, all regulated by ROS [59]. For instance, the promotion of megakaryocytic maturation requires ROS, the regulation of which is under NF-E2 p45 and not NRF2, since it activates antioxidative machinery to a lesser extent, thus enabling higher ROS levels needed for full activation of platelet genes [60].

## 4. ROS in Carcinogenesis

**Figure 2:**
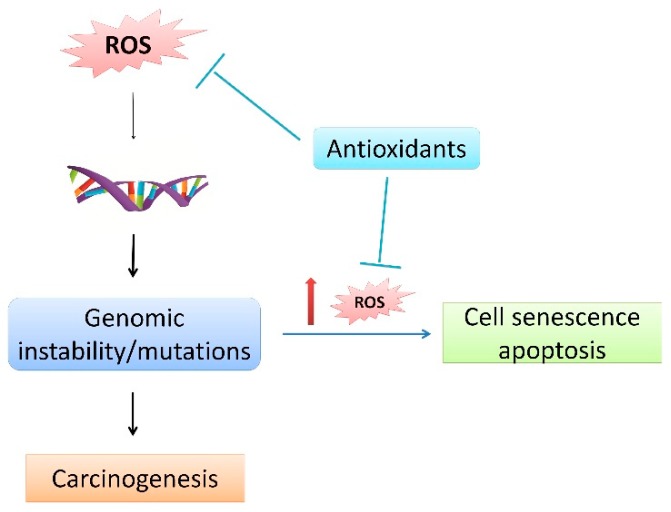
Procarcinogenic and anticarcinogenic roles of ROS.

Although the relationship between ROS and cancer has been studied for many years, even nowadays rising insights contribute to a better understanding of the complex involvement of ROS in cancer development, as well as in therapies. ROS can affect cellular macromolecules, thus inducing genomic instability and mutations, thereby implying their role in the initiation of cancer. For example, a well-established role is in chronic inflammation, where ROS derived by myeloid cells induce epithelial mutagenesis, thus stimulating invasive growth [61]. In contrast to their procarcinogenic role, ROS also have an anticarcinogenic role because this genetic instability often elicits additional ROS, which trigger cell senescence and apoptosis, thus limiting further proliferation of the transformed cells, which therefore prevents cancer progression [62]. This cancer-suppressive role of ROS is highly dependent upon the concentration of ROS and can be abolished by antioxidants, thereby confirming their role in carcinogenesis (Figure 2). Indeed, GSH and thioredoxin, both decreasing levels of ROS below the ones that would lead to apoptosis, are implicated in different stages of carcinogenesis: GSH in cancer initiation and thioredoxin in the progression of already-established cancer [63].

**Figure 3:**
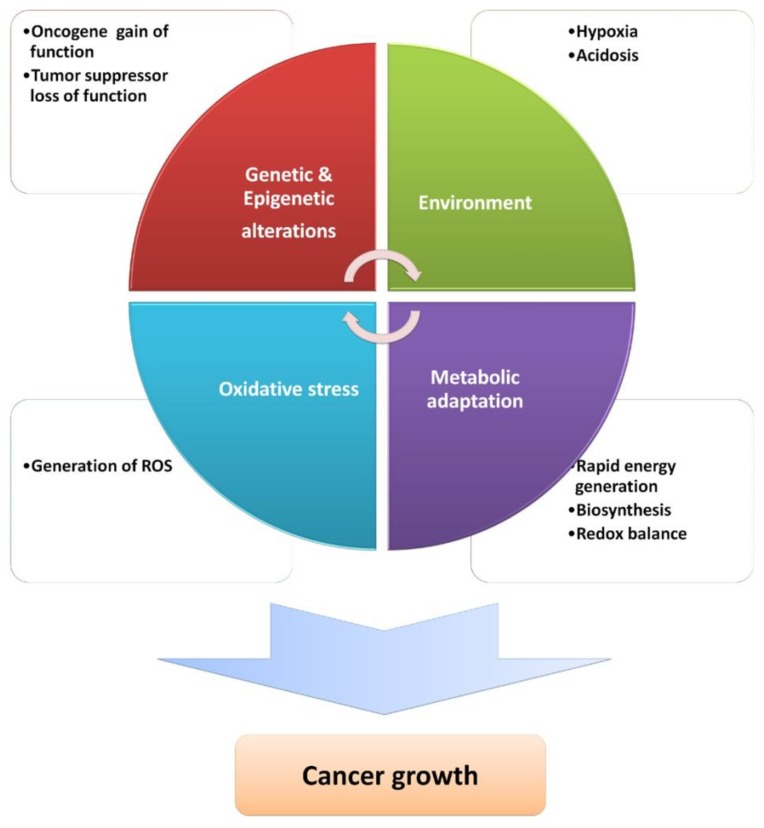
Interconnected cancer-growth-supportive alterations and factors.

Therefore, the antioxidative machinery boost, either endogenously through activation of the NRF2-KEAP1 pathway or exogenously by nutritional supplementation, is still highly controversial and seems to be dependent on the stage of carcinogenesis [7,24,62,64]. The causally consequential relationship of ROS with genetic, metabolic, and microenvironment-associated alterations occurring in cancer (Figure 3) has highlighted their importance in almost all hallmarks of cancer (reviewed in [65]). Here, we focus on the interplay between ROS, metabolic, and microenvironment-associated alterations, highlighting ROS as instruments/tools produced by cancer cells, exploiting their signaling ability to the levels contributing to cancer progression. A fine balance of ROS levels and their proximity to a specific target should be achieved to activate cancer-growth-promoting pathways such as PI3K/AKT, MAPK (Erk1/2, p38, and JNK), and IKK/NF-κB [66,67].

To provide a selective advantage, cancer cells reprogram their metabolism to support redox balance and to gain enough energy and building blocks to foster their growth [34]. Indeed, redox balance and metabolism have been closely related in reciprocal crosstalk. Metabolic alterations occurring in cancer cells include (1) a strong dependence on aerobic glycolysis known as the Warburg effect [68] (despite functional mitochondria and oxygen availability), (2) an increase in glutaminolysis, (3) activation of the PPP, (4) induction of macromolecule biosynthesis, (5) upregulation of amino acid and lipid metabolism, and (6) enhancement of mitochondrial biogenesis [69]. Although increased glycolysis is less energy productive than OXPHOS, it is preferable to OXPHOS since it generates ATP at a much faster rate, decreases potentially detrimental ROS which would be formed during OXPHOS, offers a cancer growth advantage during hypoxic and acidic conditions (lactate production via glycolysis and glutaminolysis), and intermediates for macromolecular biosynthesis [70,71]. Yet, not all cancer cells express a glycolytic phenotype, instead utilizing ATP through OXOPHOS, which suggests that their interplay is highly dependent on microenvironmental changes and demands for energy and biosynthetic activity [70,72]. Utilizing a computational systems biology approach, a core circuit containing hypoxia-inducible factor 1 (HIF 1), 5′ AMP-activated protein kinase (AMPK), and ROS was found to be robust for studying the switch between glycolysis and OXPHOS. It was thus revealed that cancer cells with the hybrid metabolic phenotype (both coexisting modes) have increased plasticity, allowing their better adaptation to microenvironmental changes and survival advantages [73]. Hence, the relationship between oncogene HIF 1, cellular energy sensor AMPK, and ROS is of immense importance for cancer evolution. Hypoxia and nutrient deprivation are side-effects of uncontrollable cancer growth, at least in the inner part of the tumor bulk. Under hypoxia, HIF 1 activates the transcription of genes responsible for glucose metabolism, angiogenesis, cell survival, and invasion [74]. Elevated levels of ROS under hypoxic conditions stabilize and activate HIF 1 [75]. An increase of ROS is also observed during glucose deprivation, leading to activation of the cellular energy sensor AMPK. The main role of AMPK is metabolic reprogramming in favor of catabolism while suppressing anabolism to promote cell survival [76]. AMPK-induced regulation of different metabolic pathways, in a feedback loop, affects ROS and tunes their levels with NADPH and GSH to those which promote survival of cancer cells [77]. Metastatic advantage upon matrix detachment was linked to Ca^2+^-ROS signaling network activation of AMPK [78]. Of note is the dual role of AMPK in carcinogenesis as a promoter of cancer cell survival under metabolic stress and as a mediator of cancer-suppressive signaling of tumor-suppressor liver kinase B1 (LKB1), respectively [79,80]. Therefore, ROS mediate metabolic reprogramming through the activation of HIF 1 and AMPK, which in turn finely tune their levels by the generation of antioxidative molecules (NADPH and GSH) as well as redox cofactors (NADH and FADH) [81]. In addition, a recent perspective suggests the metabolic reprogramming, in particular, change from glycolysis to PPP upon persistent oxidative stress as an additional dedifferentiation step from cancer cells to cancer stem cells [82].

## 5. ROS Implications in Therapy

ROS involvement in therapy strategies is best described for anticancer treatments, as most of the conventional anticancer therapy (chemo- and radiotherapy) is indeed based on excessive ROS production. In addition, the importance of ROS as a potential therapeutic tool has recently emerged in the field of regenerative medicine, which aims to heal or replace damaged cells, tissues, or organs. Therefore, these two strategies are discussed in this section.

### 5.1. ROS and Anticancer Therapy

While radiotherapy, chemotherapy, and surgery represent the majority of relatively effective anticancer treatments involving the overproduction of ROS, the use of various nutraceuticals or synthetically manufactured compounds acting directly as antioxidants or indirectly as activators of cellular antioxidative mechanisms, mainly the NRF2 pathway, is still a source of controversy in anticancer therapy [7,24]. The reason lies in the fact that higher levels of ROS induced by conventional therapies causing cancer cell death might be lowered by antioxidants, consequently promoting the survival of cancer cells. In addition, mutations in the NRF2 pathway leading to its constitutive activation were shown to be a protective mechanism in different cancers [83]. To prevent therapeutic drawbacks, it is vital to reveal the complex cancer-related redox biology considering the cancer heterogeneity, the stage of the disease, and the metabolic and redox interplay within and between cancer cells as well as with their microenvironment. It is thus crucial to define in which conditions ROS (which specifically) affect their targets (what they are) to selectively eliminate cancer cells without affecting normal cells. Since alterations in metabolic pathways differ among cancer types and are highly dependent upon cancer stage and heterogeneity, as already mentioned, the majority of conventional therapies exploit the most common selective feature of cancer cells—higher levels of ROS. Unfortunately, it is not completely selective and some unwanted side-effects can occur as a result [7,84]. Therefore, current research recognizes the advantage of combinational therapy over monotherapy. This newer approach ensures the lower adaptation ability of cancer cells and, due to the lower doses used, reduction of unwanted side-effects [85]. Keeping in mind that ROS regulate cancer cellular processes, it is important to find a switch to ROS-induced apoptosis of cancer cells. Therefore, future anticancer strategies mandate revealing the underlying beneficial mechanisms of some nutraceuticals and other anticancer compounds, especially concerning the involvement of ROS. For example, triptolide, a diterpenoid triepoxide lactone isolated from the plant *Tripterygium wilfordii* Hook F, was shown to induce apoptosis in glioma cells by the ROS-activated JNK (c-Jun N-terminal kinase) signaling pathway [86]. Another apoptosis- and autophagy-related role of the traditional Chinese herb chamaejasmine was also linked with the formation of ROS in osteosarcoma cells [87]. Recently, the mechanism underlying the anticancer properties of deoxypodophyllotoxin, a naturally occurring flavolignan isolated from *Anthriscus sylvestris*, was investigated. Growth inhibition of prostate cancer cells was linked to mitochondrial ROS-induced apoptosis and autophagy. However, the promotion of AKT-independent autophagy through ROS activation of ERK signaling was also suggested as a protective mechanism against cancer cell apoptosis. Therefore, the authors proposed usage of autophagy inhibitors, which should potentiate the observed anticancer effects of deoxypodophyllotoxin [88]. The anticancer properties of another plant-derived component, lambertianic acid, is linked with the induction of apoptosis through ROS-dependent phosphorylation of liver kinase B1/AMP-activated protein kinase/acetyl-CoA carboxylase signaling [89]. The improvement of known anticancer properties of certain naturally occurring compounds could be potentiated by synthesis of their analogs. For example, a novel analog of phenethyl isothiocyanate (an ROS-inducing and selectively cancer-cell-killing compound present in cruciferous vegetables) was suggested as a more potent ROS-modulating anticancer compound which can eliminate even cancer stem cells (a population responsible for cancer recurrence and therapy resistance) [90].

### 5.2. ROS and Regenerative Medicine

Regenerative medicine is an interdisciplinary field oriented to find the best solutions to heal or replace damaged cells, tissues, or organs in order to restore their normal functions. To do so, it involves or combines multiple approaches, such as stem cell therapy, gene therapy, tissue engineering, biomaterials, and nanotechnology. As mentioned in Section 3.1, ROS affect the main features of stem cells through the activation of diverse signaling pathways. The pharmacological regulation of ROS in stem cells was therefore suggested as a possibility for regenerative medicine [91]. Biomaterials may be needed when defects of or damages to the tissues are too great to be restored with cell therapy alone. These are synthetic or natural materials and are often made of multiple components able to interact with the biological system. Biocompatibility is a major consideration when designing biomaterials. Many studies have highlighted the importance of ROS in inflammation and healing, processes in which they act as chemoattractants and signaling molecules to recruit inflammatory and healing cells. However, their role in the biocompatibility of implanted materials may also be significant [92,93]. Since both cells and biomaterials produce ROS and are influenced by them, oxidative stress was suggested to be their most direct route of communication [92]. Oxidative stress and lipid peroxidation were shown to be activated as a growth-promoting signal in osteoblast-like cells in bioactive glasses skeletal-defect therapy [94,95]. In a recent study, modulation of the mentioned growth-promoting signal with vitamins was suggested to be beneficial for hydroxyapatite-based materials [96].

## 6. Conclusions and Future Directions

The role of ROS in physiology as well as in pathology is inevitable. Aside from their contribution to the onset of disease, when present in excess, ROS determine cellular fate, acting as signaling molecules. Not all ROS are involved in redox signaling. Hydrogen peroxide is the prominent one, but other redox messengers are involved as well. They include HNE, hydroperoxides, and thiol peroxidases [14,20]. Cellular functioning depends upon the translation of ROS-mediated messages through operating systems (target proteins) into a cell fate decision. Levels of ROS, sources of their generation, the vicinity of a target protein, and the reaction kinetics determine their redox signaling potential. NOX and, in particular, mitochondria, due to their ability to dynamically move closer to their targets, are suggested as the main sources of physiologically relevant ROS [9,97]. In addition, a recent suggestion that ROS-mediated cysteine modifications have broader regulatory functions substantiates ROS importance in cellular functioning [98].

Depending on the levels present, ROS switch on and off diverse signaling pathways, thus affecting all cellular processes, from proliferation to differentiation and apoptosis. This ROS-mediated, health-oriented regulation contributes to tissue development, normal functioning, and repair. Unfortunately, in diseases such as cancer, the growth-inducing properties of ROS are exploited by cancer cells. Of note, a novel mechanism has linked HNE inactivation of cancer-specific membrane-associated CAT with the subsequent increase of ROS and selective induction of apoptosis in cancer cells [99].

In this review, our aim was not only to state the importance of ROS in health and disease (focusing on cancer) but also to highlight the role of ROS in the regulation of how cells function within and between themselves. A close and intertwined connection of antioxidative machinery and metabolism is responsible for the fine-tuning of ROS and the elicitation of ROS-specific actions that also affect them. Thus, depending on the stimuli, cells exploit ROS signaling ability to activate specific pathways that will determine their fate.

Therefore, future research should focus not only on ROS-specific targets but also on their feedback loops with antioxidative mechanisms, other signaling molecules, and metabolic fingerprint in a cell/tissue-specific manner. In a disease state, the stage of the disease and cellular heterogeneity, as well as ROS involvement in therapeutic approaches, should be stressed to yield a better understanding of ROS involvement in physiology and pathology. Future findings should elucidate whether ROS are key regulators of cellular functions, factors affecting their formation and action, and the extent to which they can be manipulated. The understanding of these aspects might influence future therapy progress.
